# The Prognostic Value of a Tumor Microenvironment-Based Immune Cell Infiltration Score Model in Colon Cancer

**DOI:** 10.3389/fonc.2021.728842

**Published:** 2021-09-27

**Authors:** Xingkui Tang, Minling Liu, Xijun Luo, Mengyuan Zhu, Shan Huang, Xiaofen Pan

**Affiliations:** ^1^ Department of General Surgery, Guangzhou Panyu Central Hospital, Guangzhou, China; ^2^ Department of Oncology, The Seventh Affiliated Hospital, Sun Yat-sen University, Shenzhen, China

**Keywords:** colon cancer, immune cell infiltration, tumor microenvironment, prognostic biomarker, survival

## Abstract

The current study aimed to construct a prognostic predictive model based on tumor microenvironment. CIBERSORT and ESTIMATE algorithms were used to reveal the immune cell infiltration (ICI) landscape of colon cancer. Patients were classified into three clusters by ConsensusClusterPlus algorithm. ICI scores of each patient were determined by principal component analysis. Patients were divided into high and low ICI score groups. Survival, gene expression, and somatic mutation of the two groups were compared. We found that patients with no lymph node invasion, no metastasis, T1–2 disease, and stage I–II had higher ICI scores. Calcium signaling pathway, leukocyte transendothelial migration pathway, MAPK signaling pathway, TGF β pathway, and Wnt signaling pathway were enriched in the high ICI score group. Immune-checkpoint and immune-activity associated genes were decreased in high ICI score patients. Patients in the high ICI score group had better survival. Prognostic value of ICI score was independent of tumor mutational burden (TMB). The ICI score model constructed in the current study may serve as an independent prognostic biomarker in colon cancer.

## Introduction

Colorectal cancer is the third most common cancer, and it is the third leading cause of death in all cancers (1). The American Joint Committee on Cancer/Union Internationale Contre le Cancer (AJCC/UICC) TNM stage system is the most widely used prognostic predictive system (2). However, it was indicated that patients with the same TNM stage showed different clinical outcomes, revealing the defects of the TNM stage system (3). Other tumor cell characteristics were also used to classify colon cancer, including tumor morphology, tumor cell of origin, tumor gene expression, and tumor mutational status (4). But all the current cancer classifications are based on tumor cells and do not take immune status and microenvironment of tumor into consideration. The deficiency of immune system affects the ability of the body to eliminate tumor cells and promotes tumorigenesis (5). The components of tumor microenvironment, including fibroblasts, endothelia cells, cytokines, chemokines, and metabolism products, also play important roles in tumorigenesis (6, 7). A prognostic predictive model that takes the immune status and tumor microenvironment into consideration may work better in predicting prognosis.

In the current study, we constructed an immune cell infiltration (ICI) score model in colon cancer, described the somatic mutation characteristic, and validated the prognostic value of the ICI score model.

## Material and Methods

### Data Resource

Transcriptome and clinical data of colon cancer patients were downloaded from The Cancer Genome Atlas (TCGA: TCGA-COAD, https://portal.gdc.cancer.gov/repository) and Gene Expression Omnibus (GEO: GSE17536, GSE17537, GSE28722, GSE29621, GSE38832, and GSE39582, https://www.ncbi.nlm.nih.gov/geo/) database. Data were analyzed by R software (R x64 v4.0.5, https://www.r-project.org/) and Perl software (strawberry-perl, v5.32.0, https://www.perl.org/). The expression data from TCGA were transformed into transcripts per kilobase million (TPMs). The ComBat algorithm was used to decrease the batch effects between different datasets (8).

### Clustering of Immune Cell Infiltration

TCGA and GEO data were merged, and immune cell filtrations were calculated by CIBERSORT algorithm (9). Estimation of Stromal and Immune cells in Malignant Tumor tissues using Expression data (ESTIMATE) (10) package was used to calculated immune score and stromal score. Total immune microenvironment score was the sum of the stromal score and immune score. Corrplot package was used to analyze the correlation of different contents in immune microenvironment. The clustering of samples was executed using ConsensusClusterPlus algorithm (11) and was repeated 1,000 times. The ConsensusClusterPlus algorithm subsamples a proportion of items from a matrix. Each subsample is partitioned into k (range from 2 to 9) groups by a k-means algorithm. Consensus distributions for each k were displayed by cumulative distribution function (CDF) plots. The CDF plots help to find the k with an approximate maximum distribution, which indicates a maximum stability. The average consensus value between an item and the members of a cluster is measured by item-consensus (IC). The average pairwise IC of items in a consensus cluster is estimated by cluster-consensus (CLC). A bar plot is used to show the CLC values at each k.

### Analysis of Gene Expression Difference

Limma package of R software was used to compare the gene expression difference of different ICI clusters. Adjusted *p* < 0.05 and absolute fold-change >1 were considered as significant.

### Calculation of Immune Cell Infiltration Score

Based on gene expression difference, patients were classified into different clusters using unsupervised clustering. Genes that were positively and negatively related to cluster signature were assigned as ICI gene signatures A and B. Boruta package was used to performed the dimension reduction (12) of ICI gene signatures A and B. Then principal component analysis (PCA) was performed to extract the principal component 1 as signature score. The ICI score of each sample was calculated according to the formula: ICI score = ∑PC1_A_ − ∑PC1_B_ (13).

### Somatic Mutation Analysis

Nucleotide variation data were downloaded from TCGA database (https://www.cancer.gov/tcga/). The total number of non-synonymous mutation was counted. The somatic mutations of colon driver genes were evaluated using the Maftool package of R software (14).

### Statistical Analysis

The correlation between different contents was analyzed by Pearson’s test. Survival difference between different groups was analyzed by log-rank test. ICI difference and tumor mutational burden (TMB) difference were analyzed by the Wilcoxon test. Mutation difference in difference group was analyzed by chi-square test. Difference was considered to be statistically significant if *p* < 0.05.

## Result

### The Analysis of Immune Cell Infiltration in Tumor Microenvironment of Colon Cancer

A total of 1,576 cases with transcriptome information and clinical data were downloaded from TCGA and GEO database in March 2021. Patient characteristics are shown in **Supplementary Table S1**. ICI was quantified by CIBERSORT and ESTIMATE algorithms, and clustering was performed to classify the patients into different clusters (9, 10).

**Figure 1:**
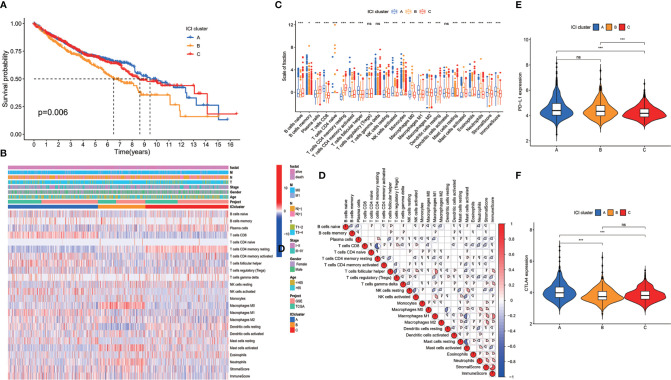
Immune cell infiltration (ICI) in tumor environment of colon cancer patients. **(A)** Colon cancer patients were classified into three clusters according to ICI in tumor microenvironment. Rows represent immune cells infiltrated in tumor microenvironment, and columns represent samples. **(B)** Overall survival of colon cancer patients in the three ICI clusters (log-rank test, *p* = 0.006). **(C)** The proportion of immune cells in three ICI clusters. The immune score and stromal score of three ICI clusters were also plotted (mean ± SD, Kruskal–Wallis test. *p < 0.05; **p < 0.01; ***p < 0.001). **(D)** Cellular interaction of the tumor-infiltrating immune cell types. **(E)** *PD-L1* expression and **(F)** *CTLA4* expression in the three ICI clusters (Kruskal–Wallis test, ***p < 0.001; ns, non significant).

The CDF plots showed that the stability increased with the increase of k. However, CLC decreased with the increase of k (**Supplementary Figure S1**). When k = 3, CDF was significantly higher than when k = 2, but when k was equal to or greater than 4, the increase of CDF was little, but the decrease of CLC was large (**Supplementary Figure S1**). Thus, we classified the patients into three clusters based on CDF and CLC. The patients were classified into ICI cluster A, ICI cluster B, and ICI cluster C. Survival between the three ICI clusters was significantly different. ICI cluster A showed the best survival, while ICI cluster B showed the worse survival (*p* = 0.006) (**Figure 1A**). We made a further comparison of the immune cell contents in tumor microenvironment of the three different ICI clusters. ICI cluster A showed high infiltration of CD8 T cell, follicular T helper cells, activated memory CD4 T cells, activated NK cells, and M1 macrophages. ICI cluster B showed a lower infiltration of CD8 T cell, follicular T helper cells, activated memory CD4 T cells, activated NK cells, and M1 macrophages but had a higher level of resting NK cells, M0 macrophages, and activated mast cells (**Figures 1B, C**). We also made a correlation coefficient heatmap to visualize the immune cell interaction in tumor microenvironment. It was shown that immune score was positively related with stromal score, neutrophils, eosinophils, resting mast cells, activated dendritic cells (DCs), macrophages, activated NK cells, γδT cells, and follicular helper T cells and negatively related to active mast cells, resting DCs, resting NK cells, regulatory helper T cells, resting memory CD4+ T cells, and plasma cells (**Figure 1D**). The expressions of two important immune checkpoint molecules, *PD-L1* and *CTLA4*, were examined. Both *PD-L1* and *CTLA4* expressions were higher in ICI cluster A than ICI cluster C. But the expression of *PD-L1* did not show a difference between ICI cluster A and ICI cluster B. The expression of *CTLA4* did not show a difference between ICI cluster B and ICI cluster C (**Figures 1E, F**). The survival difference of the three clusters may be related to the level of tumor-infiltrating immune cells and the expression of immune checkpoint molecules.

### Immune Gene Cluster Analysis

**Figure 2:**
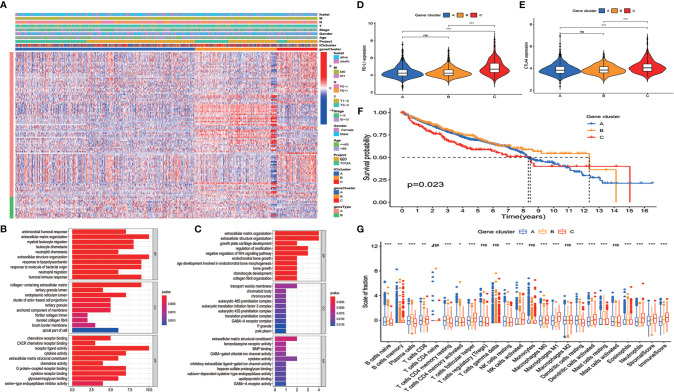
Clustering of immunogenic gene. **(A)** An unsupervised clustering of the differentially expressed genes was used to classify colon cancer patients into three gene clusters: gene cluster A, gene cluster B, and gene cluster C. **(B, C)** Enriched biological processes in immune cell infiltration (ICI) signature A **(B)** (*p* < 0.05) and signature B **(C)** (*p* < 0.05). **(D)** *PD-L1* and **(E)** *CTLA4* expressions in the three gene clusters (Kruskal–Wallis test, ***p < 0.001). **(F)** Overall survival for patients in the three gene clusters (the log-rank test, *p* = 0.023). **(G)** The proportion of immune cells in three gene clusters. The immune score and stromal score of three ICI clusters were also plotted (mean ± SD, Kruskal–Wallis test. *p < 0.05; **p < 0.01; ***p < 0.001; ns, non significant).

To reveal the gene expression characteristics in different ICI clusters, we used the limma packages of R software to analyze the transcriptome variations (**Supplementary Table S2**). Then an unsupervised clustering of the differentially expressed genes was performed. Patients were classified into three gene clusters—gene cluster A, gene cluster B, and gene cluster C—corresponding to ICI clusters A, B, and C. Genes that were positively related to the gene cluster were assigned as ICI signature A, and the rest of the differentially expressed genes were assigned as ICI signature B (12) (**Supplementary Table S3**). A heatmap was used to visualize the transcriptomic profile difference between the three gene clusters (15) (**Figure 2A**). Gene Ontology analysis was performed to clarify the enriched biological processes in ICI signature A and signature B. The results showed that biological process including response to antimicrobial humoral, lipopolysaccharide, molecule of bacterial origin and humoral immune, extracellular matrix organization, and immune cell migration were enriched in ICI signature A. Pathways correlated with some cellular components were also enriched in ICI signature A, like collagen-containing extracellular matrix, tertiary granule lumen, endoplasmic reticulum lumen, cluster of actin-based cell projections, tertiary granule, anchored component of membrane, collagen trimer, brush border membrane, and apical part of cells. Some chemokine receptor binding pathways, cytokine activity related pathways, and cytokine receptor binding pathways were also enriched in ICI signature A (**Figure 2B**). Pathways related to bone growth, extracellular matrix organization, negative regulation of Wnt signaling pathway, chromosome component, preinitiation complex component, extracellular matrix structural constituent, some ion-channel activity, and cytokine activity were enriched in ICI signature B (**Figure 2C**). The signal pathways enriched in ICI signature A were most related to immune response or immune cells, while pathways enrich in ICI signature B were less related to immune response and immune cells. The expressions of *PD-L1* and *CTLA4* in the three gene clusters were examined. Both *PD-L1* and *CTLA4* expressions were lower in gene cluster B than ICI cluster C. But no significant difference was observed between gene cluster B and cluster A (**Figures 2D, E**). Gene cluster C had a higher expression level of *PD-L1* and *CTLA4* than gene cluster B and showed worse survival than gene cluster B. The expression level of *PD-L1* and *CTLA4* in gene clusters A and B did not show a difference, and the survival of gene cluster A and cluster B showed no difference, too (**Figures 2D–F**). The results suggested that the expression level of immune checkpoint may related to overall survival. Gene cluster C had higher infiltration of CD8 T cells, M1 macrophages, DCs, and NK cells than gene cluster B. And immune score and stromal score in cluster C were also higher than those in cluster B. However, gene cluster B showed a higher level of B cells and plasma cells and a lower level of M1 macrophages (**Figure 2G**).

### Construction of Immune Cell Infiltration Score Model

**Figure 3:**
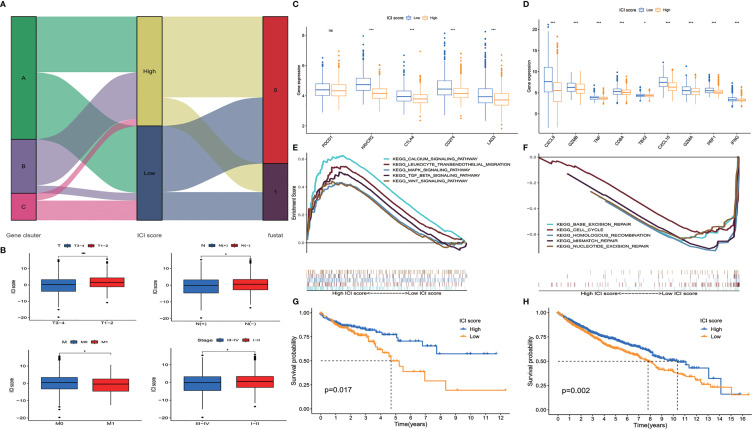
Construction of immune cell infiltration (ICI) score model. **(A)** Alluvial diagram of patient distribution. **(B)** ICI scores of patients in different subgroups (mean ± SD, Kruskal–Wallis test, *p < 0.05; ***p < 0.001). **(C)** Immune-checkpoint associated genes (*PDCD1*, *HAVCR2*, *CTLA4*, *CD274*, and *LAG3*) and **(D)** immune-activity associated genes (*CXCL9*, *GZMB*, *TNF*, *CD8A*, *TBX2*, *CXCL10*, *GZMA*, *PRF1*, and *IFNG*) expressed in ICI high and low groups (mean ± SD, Kruskal–Wallis test, *p < 0.05; ***p < 0.001). **(E)** Gene set enrichment analysis (GSEA) showed that calcium signaling pathway, leukocyte transendothelial migration pathway, MAPK signaling pathway, TGF β pathway, and Wnt signaling pathway were enriched in high ICI score group. **(F)** Base excision repair pathway, cell cycle pathway, homologous recombination pathway, mismatch repair pathway, and nucleotide excision repair pathway were enriched in low ICI score group. **(G)** Overall survival of colon cancer patients in ICI high and low groups in the Cancer Genome Atlas (TCGA) cohort (log-rank test, *p* = 0.017). **(H)** Overall survival of colon cancer patients in ICI high and low groups in the total patient cohort (log-rank test, *p* = 0.002; ns, non significant).

ICI scores of each patient were calculated by PCA. The optimal cutoff value was found out using the cutoff package of R software. Patients from TCGA database were assigned to the high ICI score group or low ICI score group according to their ICI scores (**Figure 3A**). We compared the ICI scores of different subgroup patients. It was indicated that ICI score was higher in patients who had no lymph node invasion and no metastasis. Patients with T1–2 and stage I–II disease also showed a higher ICI score (**Figure 3B**).

We evaluated the expression level of immune-checkpoint and immune-activity associated genes. *PDCD1*, *HAVCR2*, *CTLA4*, *CD274*, and *LAG3* were selected as immune-checkpoint associated genes; and *CXCL9*, *CXCL10*, *GZMB*, *GZMA*, *TNF*, *CD8A*, *TBX2*, *PRF1*, and *IFNG* were selected as immune-activity associated genes (16–18). We found that most of the immune-checkpoint genes and immune-activity associated genes, except *PDCD1*, were significantly decreased in the high ICI score group (**Figures 3C, D**). Gene set enrichment analysis (GSEA) was performed, and it was revealed that calcium signaling pathway, leukocyte transendothelial migration pathway, MAPK signaling pathway, TGF β pathway, and Wnt signaling pathway were enriched in the high ICI score group, while base excision repair pathway, cell cycle pathway, homologous recombination pathway, mismatch repair pathway, and nucleotide excision repair pathway were enriched in the low ICI score group (**Figures 3E, F**, *p* < 0.05). To evaluate the prognostic value of the ICI scores, the Kaplan–Meier analysis was performed in TCGA cohort. Patients in the high ICI score group showed better survival than patients in the low ICI group (*p* = 0.017) (**Figure 3G**). We did a further validation of the prognostic value of the ICI score in the all patients from both TCGA and GEO cohorts. It was revealed that the high ICI group also showed a better OS than the low ICI group in the total patient cohort (*p* = 0.002) (**Figure 3H**).

### The Relationship Between Immune Cell Infiltration Score and Tumor Mutational Burden

**Figure 4:**
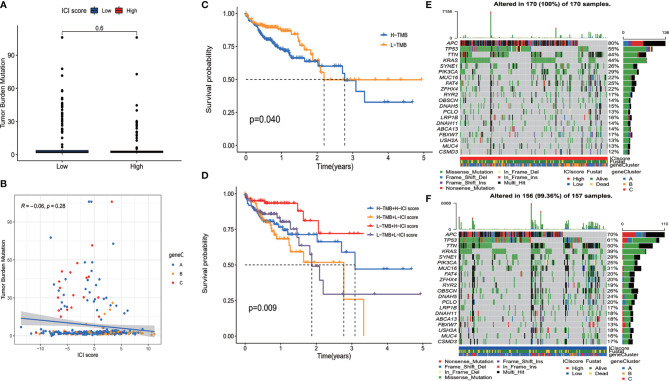
The relationship between immune cell infiltration (ICI) score and somatic mutation. **(A)** There was no significant difference on tumor mutational burden (TMB) between the two group patients. **(B)** Correlation analysis revealed that ICI score was not correlated with TMB. **(C)** Overall survival of colon cancer patients with high and low TMB (log-rank test, *p* = 0.040). **(D)** Overall survival for colon cancer patients stratified by ICI score and TMB (log-rank test, *p* = 0.009). **(E, F)** The top 20 genes with the highest mutation frequency in ICI high **(E)** and low **(F)** group. Rows represent genes, and columns represent samples.

It has been indicated that TMB is associated with patients’ response to immunotherapy (19, 20). Patients with high TMB showed an improved response to PD-1 inhibitors and other antitumor therapies. High TMB also predicted better prognosis in colorectal cancer patients (21, 22). Thus, an exploration was performed to analyze the correlation between ICI score and TMB. We compared the TMB level of patients in high and low ICI score groups. It was shown that there was no significant difference on TMB between the two groups of patients (**Figure 4A**). Correlation analysis revealed that ICI score was not correlated with TMB (**Figure 4B**). Survival analysis demonstrated that patients with high TMB had better OS than patients with low TMB (*p* = 0.040) (**Figure 4C**). To find out whether the combination of TMB and ICI score can better predict the prognosis of colon cancer patients, a stratified survival analysis was performed. Survival differences were observed between high ICI score and low ICI score patients both in high TMB and low TMB subgroups (**Figure 4D**). The above results suggested that ICI score is independent of TMB and might serve as a potential prognostic biomarker in colon cancer patients.

### Distribution of Somatic Mutation in High and Low Immune Cell Infiltration Score Groups

The distribution of somatic mutation of driver genes in colon cancer was evaluated in both high and low ICI score groups. The driver genes were evaluated using the maftools package of R software (14). We further analyzed the top 20 genes with the highest mutation frequency, and results are shown in **Figures 4E, F**. There were a total of 133 genes with significantly different mutation frequencies between high and low ICI score groups (**Supplementary Table S4**). The results might provide information for further studying the mechanism of ICI and gene mutation in immune therapy.

## Discussion

Tumor-infiltrating immune cells are important components of tumor microenvironment, and ICI is associated with tumor prognosis (13). However, most of the current prognostic assessment systems in colon cancer do not take tumor microenvironment or ICI into account.

It has been pointed out that tumor-infiltrating immune cells offered important prognostic information in cancer patients. It has been indicated that tumor-infiltrating immune cells were a prognostic biomarker in some solid cancer, including lung cancer (23), head and neck squamous cell carcinoma (24), and breast cancer (25). A prognostic immune risk score (pIRS), which was constructed based on tumor-infiltrating immune cells, was proved to be an independent prognostic biomarker for relapse-free survival in patients with resectable colon cancer (26). However, the risk score model in this study was constructed based on GEO database. The ICI score model we constructed in the current study was constructed based on both TCGA and GEO database. The data in the current study are more complete and reliable. In addition, pIRS model only predicts the postoperative recurrence risk, while ICI model in the current study predicts the overall survival risk. Basing on CD3+ and cytotoxic CD8+ T cells in a tumor, another study constructed an immune score system and revealed that the immune score system provides reliable information on the risk of recurrence in patients with colon cancer (27). However, the infiltration level of CD3+ and CD8+ T cells cannot reflect the immune status of tumor microenvironment.

In the current research, we constructed an ICI score model based on tumor immune environment using bioinformatics analysis. The current analysis included 22 types of immune cells. The ICI score model developed in this study can evaluate the prognosis of colon cancer patients to some extent. The ICI score was independent of TMB. The combination of ICI score and TMB worked better than TMB alone in assessing prognosis.

Our analysis demonstrated that ICI cluster with increased infiltration of CD8+ T cell, CD4+ T cells, activated NK cells, M1 macrophages, and ICI score had a better prognosis. The result was consistent with the previous study (28, 29). We analyzed the gene characteristics of different ICI clusters and classified the patients into three gene clusters. Though gene cluster C had higher immune score and stromal score than cluster B, patients in gene cluster B showed the better OS. There may be several reasons. Firstly, although gene cluster C had higher infiltration of T cells, gene cluster B had higher infiltration of B cells and plasma cells. Secondly, other components in tumor microenvironment, such as inflammatory factors and metabolism products, also contribute to the antitumor immune response of the host. The abnormal alteration of these molecules during tumor development may affect the interaction between the immune cells and break the balance between immunity tolerance and immunity activity (30).

Due to individual differences of ICI, it is very important to make an individual immune score for each patient. In this study, we constructed an ICI score model using the Boruta algorithm. GSEA revealed that calcium signaling pathway, leukocyte transendothelial migration pathway, MAPK signaling pathway, TGF β pathway, and Wnt signaling pathway were enriched in the high ICI score group. A previous study also indicated that hyperactivated of calcium signaling pathway and MAPK signaling pathway was associated with high infiltration of immune cell (28). Although it was indicated that TGF β was a suppressor factor to antitumor immunity, TGF β plays an important role in suppressing tumor development by inhibiting cell cycle progression, promoting apoptosis, and decreasing expression of growth factor, cytokine, and chemokine (31). Wnt signaling pathway also has dual effects on antitumor immunity. On the one hand, Wnt signaling executes an adverse effect on antitumor immunity by suppressing the effect of T-cell differentiation, inhibiting expansion of CD8+ T cells, promoting M2-like polarization of TAM, etc. On the other hand, Wnt signaling promotes DC maturation and activation, and promoting the trafficking of DC and T cells to tumor tissue (32).

TMB was regarded as a predictive biomarker of immunotherapy. We also evaluated the TMB in the current study and found that patients with high TMB showed better survival. There was no significant difference on TMB between high and low ICI score group patients. Besides, there was no correlation between TMB and ICI score. ICI score and TMB represented different aspects of tumor immunity, and ICI score was a prognostic biomarker independent of TMB in colon cancer.

In conclusion, we analyzed the ICI landscape and provided an insight on the antitumor response regulation in colon cancer. And we constructed an ICI score model that might serve as a potential prognostic biomarker independent of TMB. However, it should be noted that the ICI score model was constructed and validated in colon cancer patients lacking information about their treatment. For patients who received specific treatment, such as surgery or immunotherapy, the predictive value of this ICI score system needs further validation.

## Data Availability Statement

The original contributions presented in the study are included in the article/**Supplementary Material**. Further inquiries can be directed to the corresponding authors.

## Author Contributions

XT, ML, XL, MZ, SH, and XP had full access to all the data in the study and take responsibility for the integrity of the data and the accuracy of the data analysis. XT, ML, XL, MZ, SH, and XP designed the study. Acquisition of data: XT, ML, and XL. Analysis and interpretation of data: XT, SH, and XP. Statistical analysis: XT, SH, and XP. Drafting of the manuscript: XT, SH, and XP. All authors contributed to the article and approved the submitted version.

## Conflict of Interest

The authors declare that the research was conducted in the absence of any commercial or financial relationships that could be construed as a potential conflict of interest.

## Publisher’s Note

All claims expressed in this article are solely those of the authors and do not necessarily represent those of their affiliated organizations, or those of the publisher, the editors and the reviewers. Any product that may be evaluated in this article, or claim that may be made by its manufacturer, is not guaranteed or endorsed by the publisher.

## Supplementary Material

SUPPLEMENTARTY FIGURE 1 Cumulative distribution function (CDF) plots and bar plot of cluster-consensus (CLC). **(A)** CDF plot **(B)** bar plot of CLC.
