# Supplementary figures and images for: The Prognostic Value of a Tumor Microenvironment-Based Immune Cell Infiltration Score Model in Colon Cancer
Source: Front Oncol. 2021 Sep 27;11:728842. doi: 10.3389/fonc.2021.728842 (PMC8561118; doi:10.3389/fonc.2021.728842)

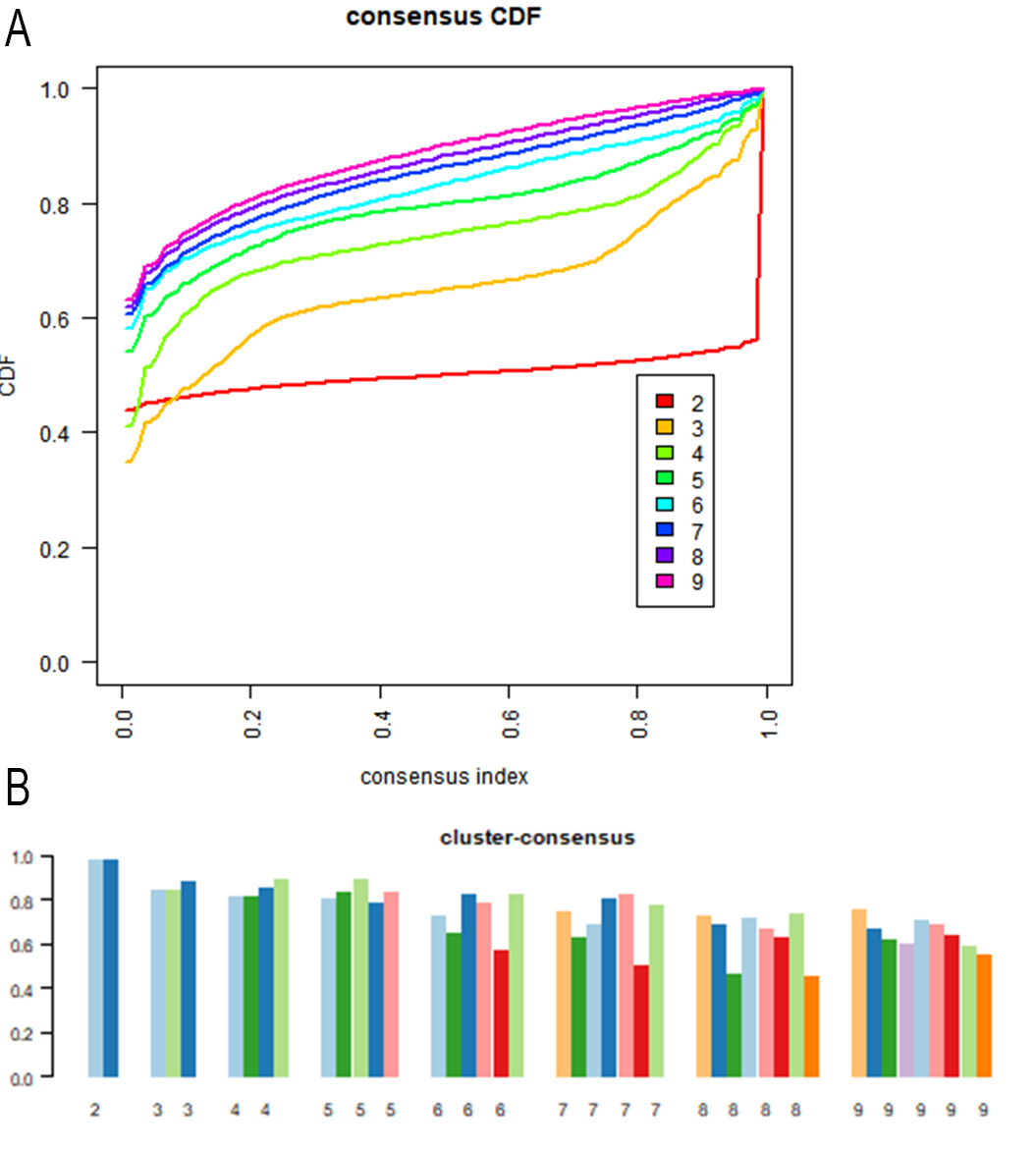

Supplement: SUPPLEMENTARTY FIGURE 1 — Cumulative distribution function (CDF) plots and bar plot of cluster-consensus (CLC). (A) CDF plot (B) bar plot of CLC. [file Image_1.tif]
